# Right Ventricular Functional Improvement After Lung Transplantation and Adjunctive Pulmonary Rehabilitation: An Echocardiographic Analysis

**DOI:** 10.3390/jcm15020437

**Published:** 2026-01-06

**Authors:** Meltem Altınsoy, Deniz Çelik, Fadime Bozduman Habip, Pınar Ergün, Hasret Gizem Kurt, Sertan Bulut, Hüsnü Baykal, Yusuf Taha Güllü

**Affiliations:** 1 Etlik City Hospital, 06170 Ankara, Turkey; meltemaltinsoy@hotmail.com; 2 Faculty of Medicine, Alanya Alaaddin Keykubat University, 07425 Antalya, Turkey; 3 Dr. Abdurrahman Yurtaslan Oncology Hospital, 06200 Ankara, Turkey; drfadimebozduman@gmail.com; 4 Atatürk Sanatoryum Research and Training Hospital, 06290 Ankara, Turkey; pinarerg@hotmail.com (P.E.); drsertanbulut@hotmail.com (S.B.); drhusnubaykal@hotmail.com (H.B.); 5 Ankara Özel Bilgi Hospital, 06370 Ankara, Turkey; gizemdr@gmail.com; 6 Vocational School, Mudanya University, 16940 Bursa, Turkey; tahagullu@gmail.com

**Keywords:** cardiopulmonary rehabilitation, lung transplantation, strain echocardiography, right ventricle

## Abstract

**Background:** Right ventricular (RV) dysfunction is common in advanced lung disease due to chronic pressure overload and altered pulmonary vascular mechanics. Lung transplantation (LTx) reduces RV afterload, and pulmonary rehabilitation (PR) may further enhance functional recovery. However, the combined effects of LTx and structured PR on RV myocardial deformation—particularly using speckle-tracking echocardiography (STE)—remain insufficiently characterized. **Methods:** This single-arm pre–post study included 20 bilateral lung transplant recipients who completed an 8-week, twice-weekly supervised outpatient PR program. Echocardiographic evaluation—including 2D measurements, M-mode, tissue Doppler imaging (TDI), and STE-derived strain parameters—was performed immediately post-discharge (baseline) and after PR. RV global longitudinal strain (RVGLS) and RV free-wall longitudinal strain (RVFWS) served as primary functional outcomes. **Results:** Improvements were observed in RV myocardial deformation after PR. RVGLS improved from a median of 15.52% to 16.64% (*p* = 0.004), and RVFWS increased from 15.82% to 17.10% (*p* = 0.001). RV mid-cavity diameter decreased significantly (*p* = 0.042), reflecting favorably altered RV geometry. Conventional parameters—including TAPSE, S′ velocity, RVEDA, and FAC—showed no statistically significant changes. These findings indicate that STE parameters are more sensitive than traditional indices for detecting early RV remodeling in the post-transplant period. **Conclusions:** Lung transplantation combined with a structured PR program was associated with early improvements in RV deformation indices measurable by STE, even when traditional echocardiographic indices remained within normal limits. STE may therefore serve as a sensitive tool for monitoring subclinical RV recovery after LTx and for assessing the additive benefits of PR.

## 1. Introduction

Chronic lung diseases such as bronchiectasis and COPD (chronic obstructive pulmonary disease) contribute to cardiac remodeling through persistent hypoxemia, while destruction of the pulmonary vascular bed increases pulmonary vascular resistance and reduces pulmonary arterial compliance [1]. Structural alterations in the lung parenchyma and impaired gas exchange lead to pulmonary hypertension (PH), resulting in right ventricular (RV) remodeling and hypertrophy. Although this initial remodeling is compensatory in response to increased afterload, sustained pressure overload eventually promotes maladaptive remodeling and progressive RV dysfunction [2]. Importantly, even modest elevations in mean pulmonary artery pressure (mPAP)—prior to the development of overt PH—can adversely affect RV structure and function [3,4].

Echocardiography remains the primary imaging modality for evaluating RV structure and function in clinical practice. However, comprehensive assessment of the RV is challenging when using standard two-dimensional echocardiographic techniques due to the RV’s complex crescentic geometry, extensive trabeculations, and its anatomical relationship encircling the left ventricle. These characteristics limit geometric modeling and reduce the reliability of conventional RV quantification compared with left ventricular evaluation [5,6]. Additionally, conventional echocardiography relies heavily on operator-dependent visual interpretation and provides limited assessment of radial displacement or deformation, lacking the ability to quantify myocardial shortening or twisting mechanics [7]. Diagnostic sensitivity is also limited in the early stages of RV dysfunction, where commonly used indices such as fractional area change (FAC) and tricuspid annular plane systolic excursion (TAPSE) largely reflect global systolic and basal longitudinal function, respectively [8].

Myocardial deformation imaging with tissue Doppler imaging (TDI) and two-dimensional speckle-tracking echocardiography (STE) provides a more comprehensive assessment of myocardial performance [7,9]. TDI-derived tricuspid annular systolic velocity (S′) evaluates basal longitudinal systolic function, whereas STE-derived RV global longitudinal strain (RVGLS) and RV free-wall longitudinal strain (RVFWS) quantify myocardial shortening across multiple segments, offering a more complete representation of RV systolic mechanics [10]. STE is not limited by geometric assumptions—as FAC is—nor does it extrapolate global function from a single basal site, as TAPSE does. Furthermore, STE can detect subclinical myocardial dysfunction even when conventional echocardiographic measurements appear normal [11].

Right ventricular remodeling in the post-transplant period is influenced by reduced pulmonary vascular resistance after lung transplantation (LTx) and by functional gains promoted through pulmonary rehabilitation (PR). Evaluating RV function within this combined post-operative context is therefore clinically meaningful. The aim of this study was to investigate whether an 8-week structured PR program provides benefits for RV functional improvement along with the early effects of LTx. We hypothesized that the combined impact of lung transplantation and subsequent PR would be associated with measurable improvements in RV myocardial deformation parameters using STE-based techniques.

## 2. Materials and Methods

This study was conducted in accordance with the principles of the Declaration of Helsinki. Ethical approval was obtained from the local institutional ethics committee (approval number and date: 2024-BÇEW 205 and 22 January 2025). Written informed consent was obtained from all participants prior to inclusion in the study.

During the preparation of this manuscript/study, the author(s) used no AI. The authors have reviewed and edited the output and take full responsibility for the content of this publication.

The study was a retrospective, single-arm, observational study with a pre–post design. All patients served as their own controls, with assessments conducted at baseline (post-discharge) and following completion of the PR program. Twenty adult bilateral lung transplant recipients underwent the minimum eight-week, outpatient-based PR program twice a week under direct supervision; the initiation of the program was 75 ± 15 d after LTx. Patients were evaluated with echocardiography, including Two-Dimensional (2D), Motion Mode (M-mode), Tissue Doppler Imaging (TDI), and Speckle Tracking Echocardiography (STE) two months after hospital discharge, before undergoing the pulmonary rehabilitation program, and immediately upon completion of the pulmonary rehabilitation program. Baseline demographics, primary diagnosis, and body surface area (BSA) in square meters were recorded. The patients were regularly monitored after LTx until the end of the 8-week PR program. In this study, patients were included if the 8-week PR program was successfully completed. Before and after the PR program, echocardiographic parameters were performed by two cardiac imagers who were blind to the study outcome. All patients received immunosuppressive therapy comprising tacrolimus, mycophenolate mofetil, systemic steroids, and preventive antifungal, antibacterial, and antiviral medications, consistent with the usual recommended medical regimen for liver transplant recipients [12]. None of the patients had diagnoses or infectious symptoms or surgical or immunological complications such as anastomotic problems, allograft rejection, primary graft dysfunction, or chronic systemic disease such as diabetes mellitus, hypertension, or thyroid diseases.

**Figure 1:**
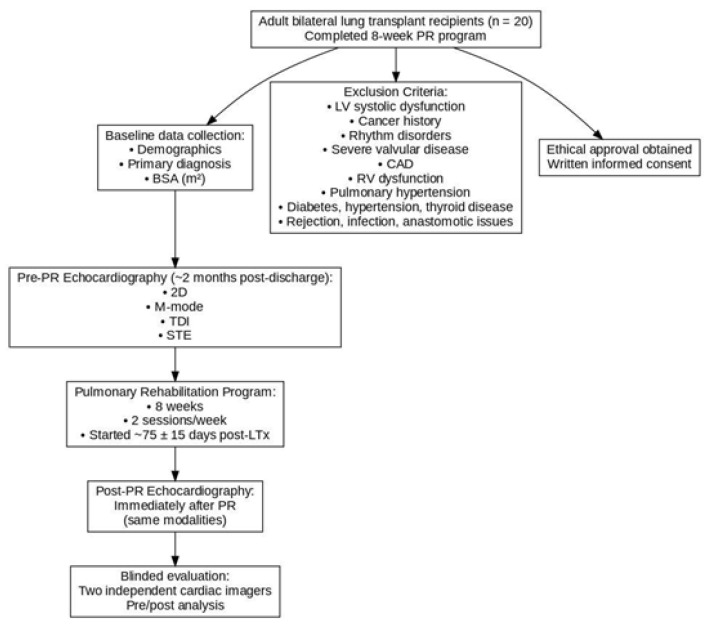
Flowchart of the study.

Moreover, patients with LV systolic dysfunction, prior cancer or existing cancer history, acute or chronic rhythm disorder, severe valvular cardiac pathologies, coronary artery disease, RV systolic dysfunction, and pulmonary hypertension were excluded due to the nature of lung transplantation candidates. An ethical approval form was taken from the local hospital committee, and a written informed consent form was delivered to patients before entering the study (Figure 1).

### 2.1. Statistical Analysis

All statistical analyses were performed using IBM SPSS Statistics for Windows, Version 27.0 (IBM Corp. Armonk, NY, USA). The normality of the continuous variables was assessed through a comprehensive, multi-parametric approach, including the following:

- Kolmogorov–Smirnov and Shapiro–Wilk tests;
- Visual inspection of histograms;
- Outlier detection via the 1.5 × IQR rule;
- Evaluation of skewness and kurtosis values.

Although this multi-method assessment was applied to evaluate distributional assumptions, due to the small sample size (*n* = 20) and to ensure consistency across variables, the non-parametric Wilcoxon signed-rank test was used for all pre- and post-pulmonary rehabilitation comparisons. Results were presented as median (interquartile range, IQR).

Descriptive statistics for baseline demographic and clinical variables were summarized using both mean ± standard deviation (SD) and median (IQR), depending on data distribution and clinical interpretability.

All statistical tests were two-tailed, and a *p*-value < 0.05 was considered statistically significant. The confidence level was set at 95% for all analyses.

### 2.2. Content of Pulmonary Rehabilitation (PR) Program

Patients participated in an 8-week, interdisciplinary, comprehensive outpatient pulmonary rehabilitation program. All patients participated in biweekly supervised fitness training sessions. Physical endurance was assessed using the 6-min walk test (6MWT) before the first session and after completing the final session of the program. Subjective dyspnea was evaluated at baseline and at the end of each session using both the Borg scale and the Medical Research Council (MRC) dyspnea scale.

### 2.3. Details of PR Program

The interdisciplinary team in PR comprises a competent pulmonologist, two physiotherapists, a dietitian, a psychologist, and a nurse. All patients seeking admission to our PR facility undergo an assessment for eligibility for the PR program conducted by the cardiologist and pulmonologist serving as the medical moderator. All patients are provided with psychological support, encompassing effective strategies for managing chronic conditions, alongside daily practical training that promotes healthy behaviors, including regular physical activity, nutritious eating, regular medication use, and adherence, as well as disease self-management. Patients in need of medical care are evaluated by a psychiatrist. Every case undergoes nutritional evaluation with the dietitian based on a body composition assessment and receives nutritional supplements if required. Additionally, training and relaxation exercises are given to relieve shortness of breath. Then, in line with the clinician’s recommendations, the patients are evaluated in terms of exercise capacity and muscle strength by the physiotherapist. Subsequent to these systematic assessments, a tailored PR program was developed. Patients engaged in an eight-week, hospital-based outpatient pulmonary rehabilitation program, attending sessions twice weekly under direct supervision. The endurance training comprised 30 min of exercise, with 15 min on a treadmill and 15 min on a stationary bike, first at 75–80% and subsequently increasing to 85% of each patient’s VO2 peak, as assessed with the Incremental Shuttle Walk Test (ISWT). As the muscle strength of the patients improved session by session, the exercise intensity was increased to the grade they could endure. A 15-min warm-up and cool-down period was also included. During the warm-up phase, patients engaged in isotonic stretching performances, whereas the cool-down phase used specialized strategies to facilitate muscular relaxation. A single set of 10 repeats for upper extremity resistance exercise commenced with 0.5 kg and progressively escalated to 1–1.5 kg based on individual tolerance. Initial lower extremity muscle engagement began at 25–30% for one set, then increased to 50–65%. The quadriceps resistance training involved utilizing free weights biweekly for 8 weeks, commencing at 25–30% for 1 set of 10 repetitions, and escalating to 70% for 3 sets. Respiratory muscle exercise was conducted by progressively augmenting the exertion to the maximum degree of tolerance, sustained for 20–30 min daily. Physiotherapists monitored vital signs during PR sessions. The pulmonologist evaluated the symptoms throughout each session and implemented the requisite interventions. Upon completion of programs, patients underwent examinations, and all examinations from the initial assessment were re-evaluated. Alongside the supervised exercise regimen, all cases were directed to engage in a home-based exercise program once weekly. The regimen comprised breathing exercises, ambulation, and strengthening activities for both extremities. To facilitate the implementation of the home exercise program, each patient was provided with a follow-up chart, and the physiotherapist conducted follow-up assessments.

### 2.4. Echocardiographic Examination

Echocardiographic images obtained from Vivid systems (GE, Horton, Norway) utilizing a 2.5–3.5 MHz probe were evaluated with GE Echo PAC version BT12; the archived echocardiographic scans were reanalyzed off-line, and the subsequent measurements were obtained in accordance with international guidelines [9,13,14]. The 2D and M-mode echocardiographic images were obtained from traditional parasternal long-axis, short-axis, and apical four-chamber views in the supine position or with little left oblique rotation. Right atrial volume was obtained in end systole from the area length method. The right ventricular end-diastolic area (RVEDA) and end-systolic area (RVESA) were evaluated using manual planimetry, while the right ventricular fractional area change (RV FAC) was calculated using the following formula: ((RV end-diastolic area-RV end-systolic area)/RV end-diastolic area) × 100. The right ventricular end-diastolic long-axis diameter (RVED lax) is defined as the distance between the RV apex and the middle of the tricuspid valve annulus. The RV end-diastolic short-axis diameter (RVED sax) is described as the distance between the interventricular septum and the free wall of the right ventricle, measured orthogonally to the long axis at its midpoint. The tricuspid valve anulus diameter (TV anulus D) is the distance between the junctions of the septal and posterior leaflets during end-diastole. Tricuspid annular plane systolic excursion (TAPSE) is a measurement of the tricuspid annulus from the end of diastole to the end of systole by using M-mode at the lateral tricuspid annulus. Pulsed-wave TDI was employed to assess tricuspid peak annulus systolic velocity (S′). Right ventricular strain was assessed by speckle tracking echocardiographic technique with standard two-dimensional echocardiographic grayscale apical four-chamber view by using offline using a dedicated software (EchoPAC, BT12, GE Healthcare, Chicago, IL, USA) quantification software. RV global longitudinal strain results were obtained as average strain values of 6 segments (basal, mid, and apical segments of the RV free wall and septum), obtained from an apical 4-chamber view. RV free wall longitudinal strain (RVFWLS) was calculated as the mean of the RV lateral basal, mid, and apical segments, with exclusion of the septal segments. The speckle tracking study was repeated with 8 randomly selected patients after 1 month to analyze intraobserver and interobserver variability, calculated as the mean difference between the 8 measurements evaluated by the same observer or a second independent observer, respectively. In our study, the intraobserver variability was 6.1% and the interobserver variability was 8.8%.

## 3. Results

A total of 20 patients completed the pulmonary rehabilitation (PR) program. The group had a mean age of 37.5 ± 6.6 years, with equal gender distribution (10 males and 10 females). The predominant diagnosis was bronchiectasis (n = 14), followed by idiopathic pulmonary fibrosis (IPF, n = 6). Mean BMI was 22 ± 3 kg/m^2^. Baseline pulmonary function tests demonstrated a mean FEV_1_ of 75 ± 15% predicted and an FVC of 66 ± 16% predicted. The left ventricular ejection fraction was preserved (median 63%, IQR 60–65%) (Table 1).

A total of 20 lung transplant recipients underwent echocardiographic evaluation before (T0) and after (T1) a structured pulmonary rehabilitation (PR) program. The analysis focused on key right heart structural and functional parameters.

**Figure 2:**
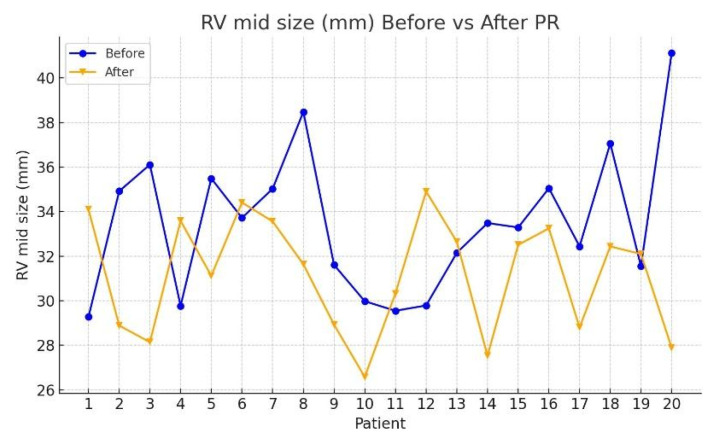
Comparison of echocardiographic parameters of RV mid-size before and after PR program.

**Figure 3:**
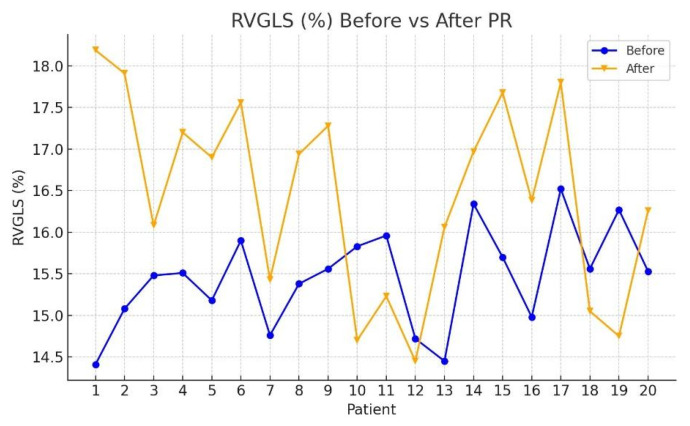
Comparison of echocardiographic parameters of RVGLS before and after PR program.

**Figure 4:**
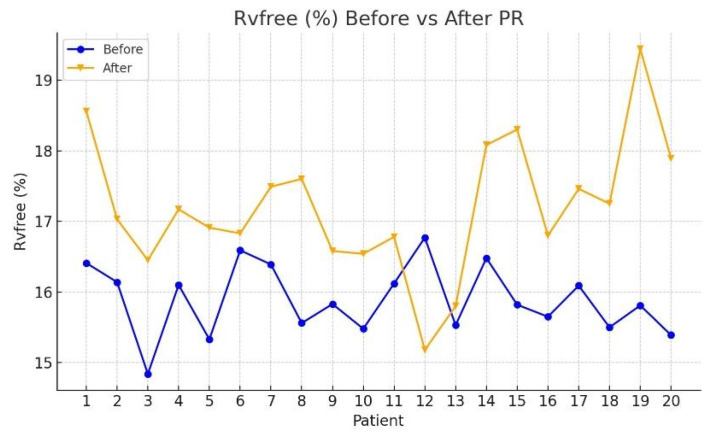
Comparison of echocardiographic parameters of RV free before and after PR program.

Table 2 presents the median values and interquartile ranges (IQR) for each echocardiographic parameter at T0 and T1, along with Wilcoxon signed-rank test results. Among the evaluated variables, significant post-rehabilitation improvements were observed in the following:

- RV mid-size: median reduced from 33.39 mm (IQR: 31.16–35.16) to 31.87 mm (IQR: 28.87–33.33); *Z* = −2.035, *p* = 0.042 (Figure 2);
- RVGLS (%): median increased from 15.44% (IQR: 14.71–15.86) to 16.46% (IQR: 15.72–17.47); *Z* = −2.856, *p* = 0.004 (Figure 3);
- RvfreeWLS (%): median increased from 15.78% (IQR: 15.36–16.29) to 16.85% (IQR: 16.39–17.48); *Z* = −3.472, *p* < 0.001 (Figure 4).

Although there were trends toward improvement in RVEDA, RVESA, and RV length, these did not reach statistical significance (*p* > 0.05). Similarly, no significant changes were detected in RA volume, TAPSE, or S′ velocity following PR.

**Figure 5:**
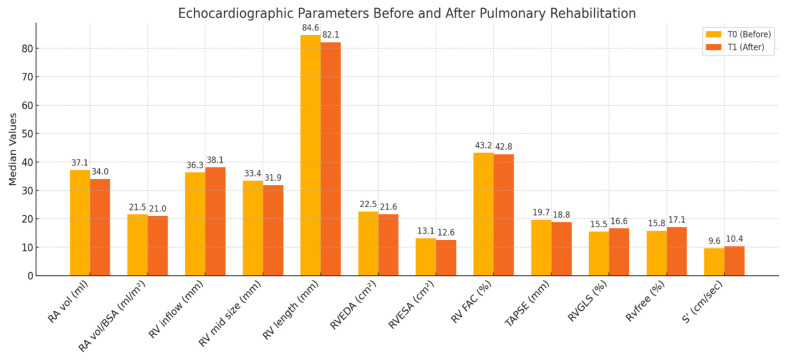
Differences over Time in Measurement Values.

A comparative bar plot (Figure 5) illustrates the changes in median values for all echocardiographic indices. Notably, functional parameters associated with right ventricular contractility and deformation (e.g., RVGLS and Rvfree) showed the most marked and statistically significant improvements.

## 4. Discussion

This study assessed the impact of a bilateral lung transplantation and adding a post-transplant 8-week outpatient pulmonary rehabilitation program, conducted biweekly under direct supervision, on the right heart function of lung transplant recipients. Although all patients demonstrated normal conventional echocardiographic measurements soon after transplantation, we found improvement in right ventricular myocardial deformation parameters even in the absence of clinical manifestations of abnormal cardiac function. Our current study demonstrates an association between LTx and the PR program’s cumulative effect on RV remodeling. This is important information since it suggests that strain-based imaging may serve as a more sensitive tool than traditional indices for detecting early RV reverse remodeling in the post-transplant period, and the PR program may contribute to intensively, time, and add different treatment processes such as RV pressure decline therapy during the time on the waiting list or the after surgical period.

These findings suggest that strain-based imaging may serve as a more sensitive tool than traditional indices for detecting early RV reverse remodeling in the post-transplant period.

Chronic lung disorders are linked to anatomical and mechanical alterations in the pulmonary vascular bed that elevate right ventricular afterload. This course of constriction and rigidity transpires in the proximal and distal pulmonary arteries, leading to elevated pulmonary vascular resistance and diminished pulmonary artery compliance. Chronic pressure overload due to pulmonary hypertension results in right ventricular hypertrophy and dilatation, ventricular septal displacement, and varying degrees of right ventricular dysfunction [15]. Even if PHT does not occur in chronic lung diseases, structural and mechanical alterations transpire in the pulmonary vascular bed, resulting in an elevation of right ventricular afterload. Hilde et al. suggest that right-sided cardiac complications commence early in the progression of pulmonary vascular disease, are cumulative, and result in right ventricular damage even at preclinical elevations of mean pulmonary arterial pressure, pulmonary vascular resistance, and diminished pulmonary artery compliance [7]. STE can serve as a diagnostic tool in the initial phases of certain cardiomyopathic disorders [16]. Ritchie et al. reported reductions in right ventricular size and enhancements in function occurring within weeks following single-lung transplantation in patients with severe pulmonary hypertension prior to transplantation [17]. Hansmann et al. reported full recovery of right ventricular systolic function after the bilateral lung transplantation of severe pulmonary arterial hypertension [18].

The literature clearly demonstrates the benefit of pulmonary rehabilitation (PR) programs. In a study where right ventricular functions were evaluated before and after pulmonary rehabilitation in patients with COPD using speckle tracking echocardiography, it was shown that PR improved right ventricular functions in a manner similar to our study, through right ventricular global longitudinal strain analysis [19]. The mechanism by which aerobic training mitigates left ventricular remodeling remains unclear; nevertheless, it may be attributed to a decrease in vasoconstrictive neurohormones or a reduction in hemodynamic loads [20]. Brum et al. observed that 8 weeks of aerobic training significantly reduced sympathetic tone and increased vagal modulation, reducing vasoconstrictive neurohormonal drive. This decreases RV afterload and may facilitate reverse remodeling and improved vascular parameters in chronic heart failure [21]. Exercise increases mitochondrial density, optimizes substrate utilization, and reduces oxidative stress. These adaptations improve RV myocardial efficiency, particularly under conditions of recovering afterload. The enhanced sympathovagal equilibrium, along with the decrease in vasoconstrictive neurohormones, improves endothelial function by enhancing nitric oxide availability and lowering systemic and pulmonary vascular resistance [22]. These changes reduce RV workload, allowing for more efficient contraction. Regular exercise training increases mitochondrial density and function and optimizes substrate metabolism in heart failure, which, in turn, can restore or improve cardiac function. It supports the idea that exercise modifies intrinsic myocardial energetics—a mechanism complementary to hemodynamic/neurohormonal effects [23,24]. Enhanced skeletal muscle efficiency reduces ventilatory demand, lowers metabolic stress, and indirectly decreases RV afterload by improving oxygen extraction and reducing ventilatory pressures [25]. Numerous outpatient-based pulmonary rehabilitation programs, conducted over 6–8 weeks with sessions occurring 2 to 3 times weekly for lung transplant recipients, adhere to standard pulmonary rehabilitation guidelines [26,27]. An 8-week pulmonary rehabilitation treatment provides greater long-term advantages for patients referred in the early stages of the condition [28]. Our study aligns with these findings, as improvements in RV mid-cavity dimensions and RV strain parameters were observed following the early post-transplant period and together with a standardized 8-week PR program.

Longitudinal dysfunction, assessed by tricuspid annular plane systolic excursion (TAPSE), serves as a prognostic indicator in pulmonary hypertension (PH); nonetheless, radial contraction is a more reliable indicator of global right ventricular function in conditions of elevated right ventricular afterload [29]. Both S′ and TAPSE are angle-dependent and solely represent the longitudinal function of the basal region of the right ventricle, disregarding the contributions of the apical and outflow tract components to right ventricular ejection. Furthermore, these quantities indicate myocardial motion rather than contraction. Right ventricular global longitudinal strain (RV GLS) is a better way to measure myocardial contractile function than traditional echocardiographic indices like TAPSE and S′. It is more sensitive, less load-dependent, and gives a more complete picture of the heart’s function. RV GLS can find subclinical dysfunction and improvement earlier and give better prognostic information. The assessment of right ventricular systolic function with standard echocardiographic methods appeared normal in some recently reported studies; abnormal values were found in the right ventricle with TDI and STE methods [30,31]. The STE method was determined to be a more sensitive parameter indicative of right ventricular function [32]. In a cardiac imaging study conducted before and after bilateral lung transplantation in children with severe pulmonary arterial hypertension, there was no significant change in the TAPSE value, which was within the normal range before the transplantation [18]. Therefore, it would be better to evaluate alternative measures to appraise RV function [14]. In our study, conventional measures such as TAPSE and S′ remained stable across the study period, yet STE parameters demonstrated significant improvement, emphasizing their greater sensitivity for detecting early myocardial recovery in LTx recipients. These findings reinforce the value of incorporating strain imaging into the routine follow-up of lung transplant recipients.

This study has several limitations. First, it employed a single-arm, pre–post design without a separate control group. Although this design allowed for within-subject comparisons, the absence of a control group limits the ability to distinguish the specific effects of pulmonary rehabilitation from other concurrent post-transplant recovery processes. However, since pulmonary rehabilitation is a routine component of standard post-transplant care in our hospital practice, withholding it for the purpose of forming a control group was neither ethically feasible nor clinically appropriate.

## 5. Conclusions

In our study, even in the absence of clinical indicators of atypical heart function, we detected subclinical RV dysfunction with STE, and we found improvement in right ventricular myocardial deformation parameters after early post-transplant and PR program. The increase in RV strain after the PR program and the continuous effect of the new transplant lung may lead to a reduction in right ventricular afterload and improvement in RV motions. In order to understand whether lung transplantation alone has a curative effect or a cumulative positive effect with the PR program, STE follow-up can be performed without including some of the patients in the PR program, but this study design may lead to depriving the control group of the positive effects of the rehabilitation program on cardiac function as well as the psychological, physical, nutritional, and physical resilience. Assessment of right ventricular function before and after the PR program is important in lung recipients and may be a marker of prognosis. Moreover, this improvement may help return to daily life and a physical activity routine. However, this will require confirmation in larger patient populations. Therefore, they can be used as bedside, reproducible, and reliable noninvasive tests to screen for further deterioration in RV function in LTx patients.

## Figures and Tables

**Table 1:** Demographic characteristics in lung transplant recipients who completed the PR program.

Parameter *	Completed PR Program (n = 20)
Gender (male/female)	10/10
Age (years, mean ± standard deviation)	37.5 ± 6.6
Prior diagnosis	Bronchiectasis (n = 14), IPF ^1^ (n = 6)
BMI ^2^ (kg/m^2^)	22 ± 3.23 (15–27)
Left ventricular ejection fraction (%)	63 (60–65)
FEV_1_ ^3^ (% predicted)	75 ± 15.76 (47–104)
FVC ^4^ (% predicted)	66 ± 16.61 (42–102)

IPF ^1^: idiopathic pulmonary fibrosis, BMI ^2^: body mass index, FEV_1_ ^3^: forced expiratory volume in the first second of expiration, FVC ^4^: forced vital capacity, * Data are mean ± SD or median (IQR).

**Table 2:** Analysis Result Regarding the Difference Between the First (T0) and Last (T1) Measurement Values.

Parameter (Unit)	Median (IQR) Before	Median (IQR) After	*Z*-Score	*p*-Value
RA vol mL (mL)	37.14 (32.44–42.47)	33.99 (30.04–39.72)	−1.083	0.279
RA vol/BSA (mL/m^2^) (mL)	21.55 (19.85–23.42)	21.00 (19.56–22.56)	−0.448	0.654
RV inflow (mm)	36.31 (34.54–39.85)	38.10 (34.78–38.91)	−0.112	0.911
RV mid-size (mm)	33.39 (31.16–35.16)	31.87 (28.87–33.33)	−2.035	0.042 *
RV length (mm)	84.64 (82.03–85.63)	82.12 (78.97–84.67)	−1.829	0.067
RVEDA (cm^2^)	22.54 (21.21–23.74)	21.61 (20.55–22.34)	−1.848	0.065
RVESA (cm^2^)	13.14 (11.65–13.89)	12.59 (11.78–13.61)	−0.336	0.737
RV FAC (mm)	43.24 (37.60–47.74)	42.77 (38.19–46.32)	−0.224	0.823
TAPSE (mm)	19.66 (19.07–20.70)	18.84 (17.92–21.31)	−0.877	0.38
RVGLS (%)	15.52 (15.05–15.85)	16.64 (15.38–17.35)	−2.856	0.004 *
RVFWS (%)	15.82 (15.52–16.20)	17.10 (16.73–17.67)	−3.472	0.001 *
S cm/s (mL/m^2^)	9.62 (9.06–10.34)	10.36 (9.88–10.93)	−1.867	0.062

RA vol: right atrial volume; BSA: body surface area; TV anulus D: tricuspid valve anulus diameter; RVED sax: right ventricular end-diastolic short-axis diameter; RVED lax: right ventricular end-diastolic long-axis diameter; RVEDA: right ventricular end-diastolic area; RVESA: right ventricular end-systolic area; RV FAC: right ventricular fractional area change; TAPSE: tricuspid annular plane systolic excursion; RVGLS: global right ventricular strain; RVFWS: free wall right ventricular longitudinal strain * significant *p* values.

## Data Availability

The data presented in this study are available on request from the corresponding author due to local laws.

## Abbreviations

The following abbreviations are used in this manuscript:

COPD	Chronic Obstructive Pulmonary Disease
FAC	Fractional Area Change
FEV	Forced expiratory volume in the first second of expiration
FVC	Forced vital capacity
GLS	Global Longitudinal Strain
HF	Heart Failure
IQR	Interquartile range
IPF	Idiopathic Pulmonary Fibrosis
ISWT	Incremental Shuttle Walk Test
LTx	Lung Transplantation
m(PAP)	Mean Pulmonary Artery Pressure
MRC	Medical Research Council
PH	Pulmonary Hypertension
PR	Pulmonary Rehabilitation
RA	Right Atrium
RV	Right Ventricular
RVEDA	Right ventricular end-diastolic area
RVED lax	Right ventricular end-diastolic long-axis diameter
RVED sax	Right ventricular end-diastolic short-axis diameter
RVESA	Right ventricular end-systolic area
RV FAC	Right ventricular fractional area change
RVFWLS	Right ventricular free-wall longitudinal strain
RVGLS	Right ventricular global longitudinal strain
S′	Tricuspid peak annulus systolic velocity
SD	Standard Deviation
STE	Speckle Tracking Echocardiography
TAPSE	Tricuspid Annular Plane Systolic Excursion
TDI	Tissue Doppler Imaging
